# Histological paraffin-embedded block: a good alternative specimen to detect the use of opiates at least 20 years ago

**DOI:** 10.1007/s11419-022-00614-0

**Published:** 2022-03-14

**Authors:** Domenico Di Candia, Michele Boracchi, Guendalina Gentile, Gaia Giordano, Riccardo Zoja

**Affiliations:** Dipartimento di Scienze Biomediche per la Salute-Sezione di Medicina Legale e delle Assicurazioni, Università degli Studi di Milano, Via L. Mangiagalli 37, 20133 Milan, Italy

**Keywords:** Heroin-related deaths, Paraffin-embedded blocks, Good alternative specimen, New extractive method, Stability for 20 years postmortem

## Abstract

**Purpose:**

Since the solely certain remnants of a performed autopsy are formalin-fixed paraffin-embedded (FFPE) samples, stored in the archives of every institute of legal medicine, we managed to extract molecules of toxicological interest from these specimens.

**Methods:**

We assessed the analysis of ten fresh liver samples collected from heroin-related deaths and then histologically processed the same samples. The embedded blocks were then extracted by means of a new extracting method and the eluates were measured. We also selected five toxicological cases of heroin-related fatalities that were examined 20 years ago, collected the toxicological result documents of the analysis that were carried out at the time and then processed the corresponding FFPE liver samples that were stored in the archives.

**Results:**

We managed to isolate heroine-related metabolites from 20-year-old paraffin-embedded blocks and calculated ratios to evaluate the performance of our new extraction.

**Conclusions:**

According to our study, it is feasible to carry out a toxicological examination on old histological samples and, therefore, this matrix can be considered as a new alternative specimen for chemical-analytical evaluations of past cases or when fresh samples are not available anymore. The new extractive method was evaluated as efficient in treating these complex, paraffin-embedded samples. It was surprising that the target compounds could be quantitated from FFPE bocks created as long as 20 years ago.

## Introduction

Forensic toxicology is a discipline involved in toxicological problems of forensic relevance. Common toxicological matrices are usually treated and to provide clear data regarding the assumption of xenobiotics; the possible accidental administration of substances and the malicious poisoning of a subject are well established and discussed in literature [1]. In some cases, when conventional matrices are not available, alternative matrices can be analyzed with the purpose of framing the toxicological situation of the subject of the study [2, 3]. Studies on unconventional matrices have been carried out on several substrates and are usually assessed because of lack of standard tissues or because of the availability of tissues that have undergone preservatives processes; in literature, toxicological analysis have been carried out on embalmed tissues [4] or on formalin-fixed samples [5]. In some cases, the only substrates that are available for analytical laboratory examinations are formalin-fixed paraffin-embedded (FFPE) samples. These samples have the great advantage of being stored in aeternum in the archives of each institute of legal medicine or pathology, and the paraffin embedding grants a perfect preservation of the tissues included. These embedded samples have revealed themselves of great value for several analysis; DNA and RNA extractions can be carried out on these samples [6, 7] as well as proteomic studies [8] and western-blot analysis [9]. Toxicological extractions on this type of samples, at this moment, have been attempted in a few cases [10–12], but a valid extractive procedure has not been established yet.

International guidelines [13] specify the type of sampling of organs and tissues that must be assessed on a corpse during autopsy examination and that are related to the different judiciary investigations that have to be carried out. In particular, all these samples that will undergo formalin fixation are collected with the purpose of being examined under a histological point of view that can result to be as fundamental in the determination of the cause of death and of the epicrisis, which is the cause of the death. For these analysis, fragments of sampled viscera are fixated in 10% buffered formalin and subsequently undergo a standardized procedure that consists of dehydration in increasing alcoholic ladder, clearing in a xylene substitute and embedding in high-fusion-point paraffin [13]. Following these procedures, the FFPE samples are cut into thin films, stained and examined with an optic microscope. Once these procedures are over, the remaining FFPE samples are indefinitely stored in the archives of the different institutes of legal medicine in compliance with Italian Laws. These samples represent an asset always available and immutable in time and, moreover, the solely biological remnants of a specific case in custody at every institute of legal medicine. Up to now, however, these samples are usually considered useful only for histological purposes or DNA extraction [6].

Toxicological analysis is usually assessed on fresh matrices sampled during autopsy examinations and subsequently refrigerated at a temperature of −20 °C. According to Italian law, these specimens must be safeguarded by the forensic toxicologists for a period of 5 years, and after this period of time the samples can be disposed. Further investigations are thus impossible of being conducted after this period of time.

To validate a new matrix as solid samples for future toxicological examinations, we structured our study in three phases. We toxicologically analyzed fresh liver samples collected from opiate-positive (morphine, morphine-3-glucuronide, morphine-6-glucuronide, 6-monoacetylmorphine) cases treating them accordingly to routine procedures for the Laboratory of Forensic Toxicology of the University of Milan. The identical matrices were then embedded in paraffin in accordance to the standard procedure of the Laboratory of Histopathology of the same university and then treated to toxicologically analyze them. The third phase was structured on samples embedded in paraffin 20 years previously, belonging to cases analyzed at the time and resulted to be positive for opiates according to historical forensic documents. This new approach represents a potential tool for detecting molecules of toxicological interest in these cases, in which standard toxicological samplings are not available, and the solely usable samples are paraffin-embedded viscera.

## Materials and methods

### Standard toxicology samples

From the whole pool of toxicological analysis requested to the Laboratory of Forensic Toxicology of the Bureau of Legal Medicine of the University of Milan, we selected ten cases that resulted to be positive for opiates. Liver specimens were collected during autopsy examination from the right lobe of the liver, as far as possible from the Glisson’s capsule [14]. The fresh samples, stored in refrigerators at − 20 °C, were then collected to assess a standard toxicological analysis. Fresh samples of liver were extracted using a solid-phase extraction (SPE) method.

### Chemicals and reagents

All the standard molecules used in the study were purchased from Sigma-Aldrich (St. Louis, MO, USA): morphine, morphine-3-glucuronide, morphine-6-glucoronide solutions (1.0 mg/mL in methanol each standard) and 6-acetylmorphine (1.0 mg/mL in acetonitrile standard solution). Deuterated internal standards (ISs) were purchased from Sigma-Aldrich as well: morphine-D_3_ (1.0 mg/mL in methanol standard solution), 6-acethylmorphine-D_3_ (1.0 mg/mL in acetonitrile), morphine-3-glucuronide-D_3_ (100 µg/mL in methanol with 0.05% of NaOH) and morphine-6-glucuronide-D_3_ (100 µg/mL methanol/water (1:1, v/v)). Solvents used in the extractive processes were purchased from Sigma-Aldrich (methanol, hydrochloric acid, chloroform, dichloromethane and ethyl acetate) and VWR Chemicals (Radnor, PA, USA) (acetone, acetonitrile, isopropanol and *n*-hexane). Buffer pH 6.88 solution was purchased from PanReac (Barcelona, Spain). For histological inclusions, absolute ethanol and xylene substitute were purchased from VWR Chemicals and high fusion point paraffin pearls Histowax® were purchased from International Medical Products (Brussel, Belgium).

### Working solutions’ preparation

IS working solution was prepared from deuterated IS stock solutions at a concentration of 1000 ng/mL in methanol. Opiates working solution was prepared from standard stock solutions at a concentration 10,000 ng/mL in methanol. Stock solutions were stored at −20 °C, while working solutions were stored at −20 °C and then left acclimatized at room temperature before sampling.

### SPE extraction

To extract possible analytes by SPE, we used a standard 12-port model vacuum manifold (Sigma-Aldrich, Supelco) with Laboport vacuum pump (KNF Neuberger GmbH, Freiburg, Germany) and 130 mg Bond Elut Certify cartridges (Varian, Palo Alto, CA, USA).

Liver samples of 0.5 g weight were finely ground into small pieces using a lancet, placed in a 10 mL test tube, spiked with 100 ng of IS mixture, and then mixed with 5 mL of pH 6.88 phosphate buffering solution. Extraction by means of SPE was then assessed according to a standardized procedure already described by the authors in a previous work [15]: cartridges were conditioned with 2 mL of methanol and 2 mL of pH 6.88 phosphate buffering solution, washed out with 2 mL of the pH 6.88 phosphate buffering solution, 1.5 mL of 0.01 M hydrochloric acid and 0.3 mL of methanol and left to dry for 30 min. Cartridges were than eluted with 2 mL of a mixture of chloroform/acetone (1:1, v/v) for acid/neutral analytes, and with 1 mL of ethyl acetate at 2% ammonia and 1 mL of a mixture of dichloromethane/isopropanol (5:1, v/v) at 2% ammonia for a basic extraction.

The obtained eluate was evaporated to dryness in a vacuum rotary evaporator, reconstituted in 100 µL of methanol and 2 µL of these final solutions was analyzed via high-performance liquid chromatography (HPLC)–tandem mass spectrometry (MS/MS).

### Preparation of new embedded samples

Identical liver samples in the cases previously used for SPE were then collected and processed according to the standard procedure of the Laboratory of Histopathology of the Bureau of Legal Medicine of the University of Milan. Parenchymal samples were fixed in 10% buffered formalin solution, dehydrated in an increasing alcoholic ladder (ethanol 80, 90, 95% and absolute alcohol in four steps) using an automatic linear tissue processor (ATP700; Histo-Line Laboratories, Milan, Italy), cleared using a xylene substitute in three steps and then embedded in high fusion point (56–58 °C) paraffin following three steps. The newly included viscera samples were then treated to prepare them for liquid-liquid extraction.

### Old embedded samples

The paraffin-embedded samples belonged to cadavers that underwent autopsy examination 20 years previously. At that time, acute narcotism by means of opiate overdose was diagnosed using gas chromatography–mass spectrometry (MS) and HPLC–MS/MS.

Among many of our histological archives of 20 years ago, we selected paraffin-embedded samples of livers collected from five cases. Each paraffin-embedded sample was grossly freed from the surrounding paraffin using a cutting edge, and a fragment of the weight of 0.5 g tissue was roughly taken out and crushed into rough powder using an exact knife.

### Liquid-liquid extraction of paraffin-embedded samples (both newly prepared and 20-year-old ones)

The rough powder obtained from the crushed liver samples was placed in 50 mL Falcon centrifuge test tubes (Fisher Scientific, Pittsburgh, PA, USA), and then 9 mL of *n*-hexane and 100 ng of the deuterated internal standard mixture in acetonitrile were added to each test tube. The tubes were then shaken on an ultrasound sonicator for 30 min at 50 °C and 50 Hz. After this procedure, the samples were placed on a Rotating Mixer Lab Sample Agitator (Benchmark Scientific, Sayreville, NJ, USA) for 4 h. Following this step, 5 mL of acetonitrile was added to the samples and the tubes were shaken once again on the rotating agitator for 30 min.

The samples were then centrifuged at 4 °C at 5000 rpm for 10 min and the liquid phase placed at the bottom of the tubes was collected. Five more milliliters of acetonitrile were added to the upper liquid samples, and the tubes were once again placed on a Rotating Mixer Lab Sample Agitator for 30 min. Following centrifuging at 4 °C for 10 min at 3500 rpm, the phase at the bottom of the tubes was separated and combined with the one collected previously. This phase was then evaporated in a vacuum rotary evaporator at 50 °C. The samples were then reconstituted in 100 µL of methanol. Two microliters of these final solutions was injected for analysis using an HPLC–MS/MS system.

### Instrumental conditions

Analysis were carried out using an HPLC–MS/MS TSQ Fortis II (ThermoFisher Scientific, San Jose, CA, USA.) with a Surveyor MS quaternary pump with degasser, a Surveyor AS autosampler, and an oven with Rheodyne valve with a 20 µL loop. For chromatographic separation, we used a reversed-phase HPLC column Thermo Scientific Hypersil Gold (50 × 2.1 mm, particle size 1.9 µm). Solvent A used for analysis (water at 0.1% formic acid and 10 mM ammonium formate) and B (methanol) represented the mobile phases utilized for the gradient. The HPLC gradient programming was: 0–1.0 min, 10% B; 3.0 min, 90% B; 5.0 min, 90% B; 5.5 min, 10% B; 10.0 min, 10% B. The flow rate was 0.5 mL/min. The MS conditions were: ionization mode, electrospray ionization; ion transfer tube temperature, 350 °C; vaporization temperature, 300 °C; electrospray tension, 3.5 kV; scanning acquisition, selected reaction monitoring (SRM).

The target molecules, ISs, product ions and collision energies are given in Table 1.

**Table 1:** Analytes, parent ions, product ions and collision energies for the current high-performance liquid chromatography–tandem mass spectrometry

Analyte	Precursor ion	Product ion	Collision energy (eV)
Morphine	286.2	165.0	41
		152.9	41
		151.9	55
Morphine-3-glucuronide + morphine-6-glucuronide	462.2	268.1	32
		165.1	41
		201.4	40
6-Acetylmorphine	328.4	210.9	26
		165.0	40
Morphine-D_3_	289.2	165.0	41
		152.9	44
Morphine-3-glucuronide-D_3_ + morphine-6-glucuronide-D_3_	465.2	289.1	33
		271.0	28
6-Acetylmorphine-D_3_	331.4	210.9	26
		165.0	40

### Validation procedure

Validation procedures were assessed according to the guidelines of the Scientific Working Group for Forensic Toxicology (SWGTOX) [16].

Calibration standards and quality controls for HPLC–MS/MS analysis were prepared spiking 0.5 g of blank liver samples finely ground with adequate amounts of analyte working solutions in a range 2.0–1500 ng/g, and we built two different calibration curves of six points each (2.0, 5.0, 10.0, 20.0, 40.0 and 80.0 ng/g and 80.0, 150, 300, 600, 1000 and 1500 ng/g) based on the peak area ratios of the analytes to the IS against nominal analyte concentration using a weighted 1/*x* ^2^ linear regression. Both curves were constructed with both extracting methods: SPE and liquid-liquid extraction. We tested the correlation over the whole range of concentrations to grant a linear regression; linearity was considered adequate when *r* ^2^ ≥ 0.990 and coefficient of variation (CV) % ≤ 10.0.

When analyte concentrations were outside the calibration range, analyses were repeated after sample dilution. To evaluate the integrity of the dilution process, five replicates of two different concentrations were analyzed: 8 ng/g (quality control (QC) low dilution, 1:10 dilution of 80 ng/g) and 100 ng/g (QC high dilution, 1:100 dilution of 10,000 ng/g). Dilution integrity was found to be robust and reproducible, being within the acceptable range for method validation.

Limit of detection (LOD) was defined using the lowest non-zero calibrator (2 ng/g): three blank liver matrices were fortified with adequate concentration of every analyte and the samples were analyzed three times to demonstrate that all criteria were met. The signal to noise (*S/N*) ratio for LOD was calculated and was equal to 151. Lower limit of quantitation (LLOQ) was defined as the value of the lowest non-zero calibrator according to the SWGTOX guidelines [16].

To evaluate a possible carryover effect, the highest point of the calibration curve (1500 ng/g) was followed by a blank matrix sample: the analysis was free from carryover and the data was confirmed in triplicate.

To evaluate selectivity, blank liver samples from ten different sources were analyzed and demonstrated the absence of interferences that could weaken the method.

Bias was calculated using three separate samples at three different concentrations (10.0, 600 and 1200 ng/g) over five runs. Bias was then calculated for each concentration and resulted to be ≤ 20%.

Within-run precision was calculated for each concentration of the analytes (low, medium and high) separately in triplicate over five runs and the value range obtained for each concentration was lower than 10.0 CV%. Between-run precision was calculated for each concentration over five runs and the values obtained were considered adequate as well.

Ionization suppression and enhancement (matrix effect) were evaluated for each analyte and the values ranged between −8.0 and  + 11.0%.

Quantitation of the compounds was assessed on characteristic product ions: *m/z* 152.9 for morphine, *m/z* 268.1 for morphine-3-glucuronide and morphine-6-glucuronide and *m/z* 210.9 for 6-acetylmorphine.

### Conditions of an old instrument used 20 years ago

Analyses were assessed at the time using an HPLC–MS/MS TSQ 7000 mass spectrometer (Thermo Finnigan, London, UK) equipped with an Agilent 1100 pump with degasser. For chromatographic separation, an HPLC column with reversed-phase Waters XTerra MS C-18 (100 × 2.1 mm i.d., particle size 3.5 µm) was used. Solvent A (water at 0.1% formic acid 10 mM ammonium formate) and B (methanol) represented the mobile phase utilized for the gradient. Solvent gradients for HPLC–MS/MS analysis were: 0–2.0 min, 5% B; 6.0 min, 90% B; 10.0 min, 90% B; 10.1 min, 5% B; 15.0 min, 5% B. The flow rate was 0.25 mL/min. The MS conditions were: ionization mode, atmospheric pressure ionization; ion transfer tube temperature, 350 °C; vaporization temperature, 300 °C; electrospray tension, 4.0 kV; scanning acquisition, selected reaction monitoring mode.

## Results

The concentrations of all the analytes in ten new samples without paraffin embedding analyzed by of HPLC–MS/MS are reported in Table 2. We calculated the ratios of the concentrations of every analyte in the liver samples analyzed by the above standard toxicological analysis to those of the identical analytes measured for the paraffin-embedded samples, which was extracted by our new procedure.

**Table 2:** Quantitative results of opiates metabolites detected from fresh postmortem liver samples collected from ten cadavers at autopsies and their newly created histological paraffin-embedded blocks

Case no	Sample type/ratio L/E	Morphine (ng/g)	Morphine-3-glucuronide + morphine-6-glucuronide (ng/g)	6-Acetylmorphine
1	Liver	1060	7630	ND
	Embedded	658	4680	ND
	Ratio L/E	1.61	1.63	ND
2	Liver	342	1940	ND
	Embedded	198	1150	ND
	Ratio L/E	1.73	1.68	ND
3	Liver	2110	18,500	Trace
	Embedded	1190	10,700	Trace
	Ratio L/E	1.77	1.73	ND
4	Liver	5370	26,300	Trace
	Embedded	2950	14,500	Trace
	Ratio L/E	1.82	1.81	ND
5	Liver	189	1150	ND
	Embedded	112	690	ND
	Ratio L/E	1.69	1.67	ND
6	Liver	1040	10,200	ND
	Embedded	581	5700	ND
	Ratio L/E	1.79	1.79	ND
7	Liver	1200	9840	Trace
	Embedded	740	6040	ND
	Ratio L/E	1.62	1.63	ND
8	Liver	553	3600	ND
	Embedded	327	2120	ND
	Ratio L/E	1.69	1.70	ND
9	Liver	110	4950	ND
	Embedded	634	2930	ND
	Ratio L/E	1.74	1.69	ND
10	Liver	541	4100	ND
	Embedded	304	2360	ND
	Ratio L/E	1.78	1.74	ND

*ND* not detected, *L/E* liver/embedded

The concentrations of analytes measured by toxicological analysis at the time of the first toxicological examination (20 years ago), which was listed in the old document, were compared with the concentrations of the substances in the 20-year-old paraffin-embedded samples, but were processed with our new extractive method, and their ratios are summarized in Table 3.

**Table 3:** Analytical results of hepatic opiate metabolites recorded 20 years ago and those obtained by crashing the corresponding 20-year-old paraffin-embedded blocks in this study

Case no	Recorded result/result from old block/concentration ratio	Morphine (ng/g)	Morphine-3-glucuronide + morphine-6-glucuronide (ng/g)	6-Acetylmorphine
1	Liver	1030	5900	ND
	Embedded	651	3760	ND
	Ratio L/E	1.58	1.57	ND
2	Liver	2080	12,600	Trace
	Embedded	1500	8780	ND
	Ratio L/E	1.38	1.44	ND
3	Liver	790	5560	ND
	Embedded	497	3480	Trace
	Ratio L/E	1.60	1.60	ND
4	Liver	195	1020	ND
	Embedded	121	635	Trace
	Ratio L/E	1.61	1.60	ND
5	Liver	3450	28,000	Trace
	Embedded	2350	19,200	Trace
	Ratio L/E	1.47	1.46	ND

For abbreviations, see the footnote of Table 2

**Fig. 1:**
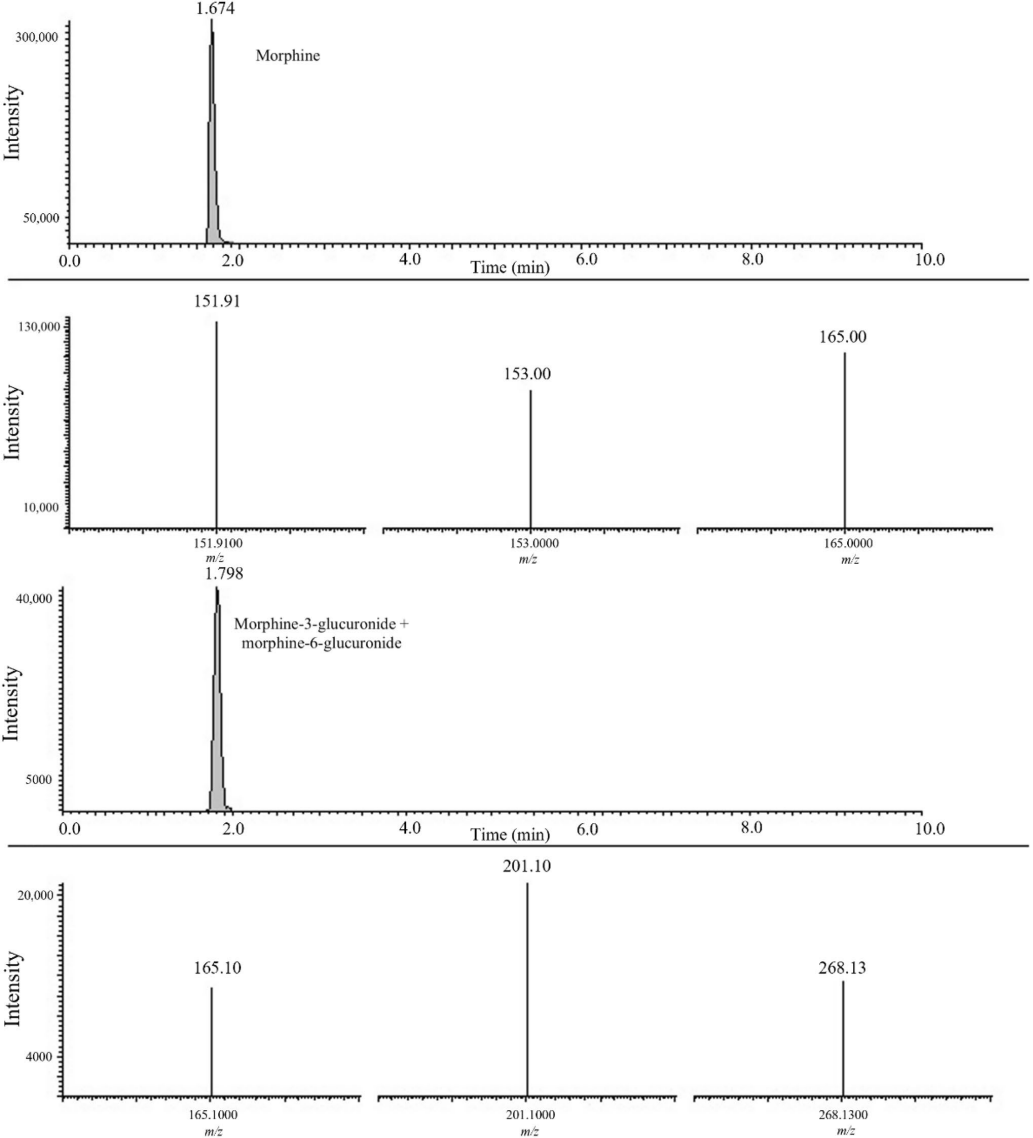
Selected reaction monitoring (SRM) chromatograms highlighting morphine (top row) and morphine-3-glucoronide + morphine-6-glucuronide (third row) and their respective product ion spectra detected from a freshly embedded paraffin block belonging to case no. 8

Morphine, morphine-3-glucuronide and morphine-6-glucuronide were detected in ten postmortem livers and freshly embedded samples selected, while 6-acetylmorphine was detected only in three cases (case nos. 3 and 4 in both liver and embedded samples and in case no. 7 only in the liver) (Table 2). SRM chromatograms of a freshly embedded sample (case no. 8) reporting morphine and morphine-3-glucuronide + morphine-6 glucuronide together with their characteristic fragmentation are shown in Fig. 1.

**Fig. 2:**
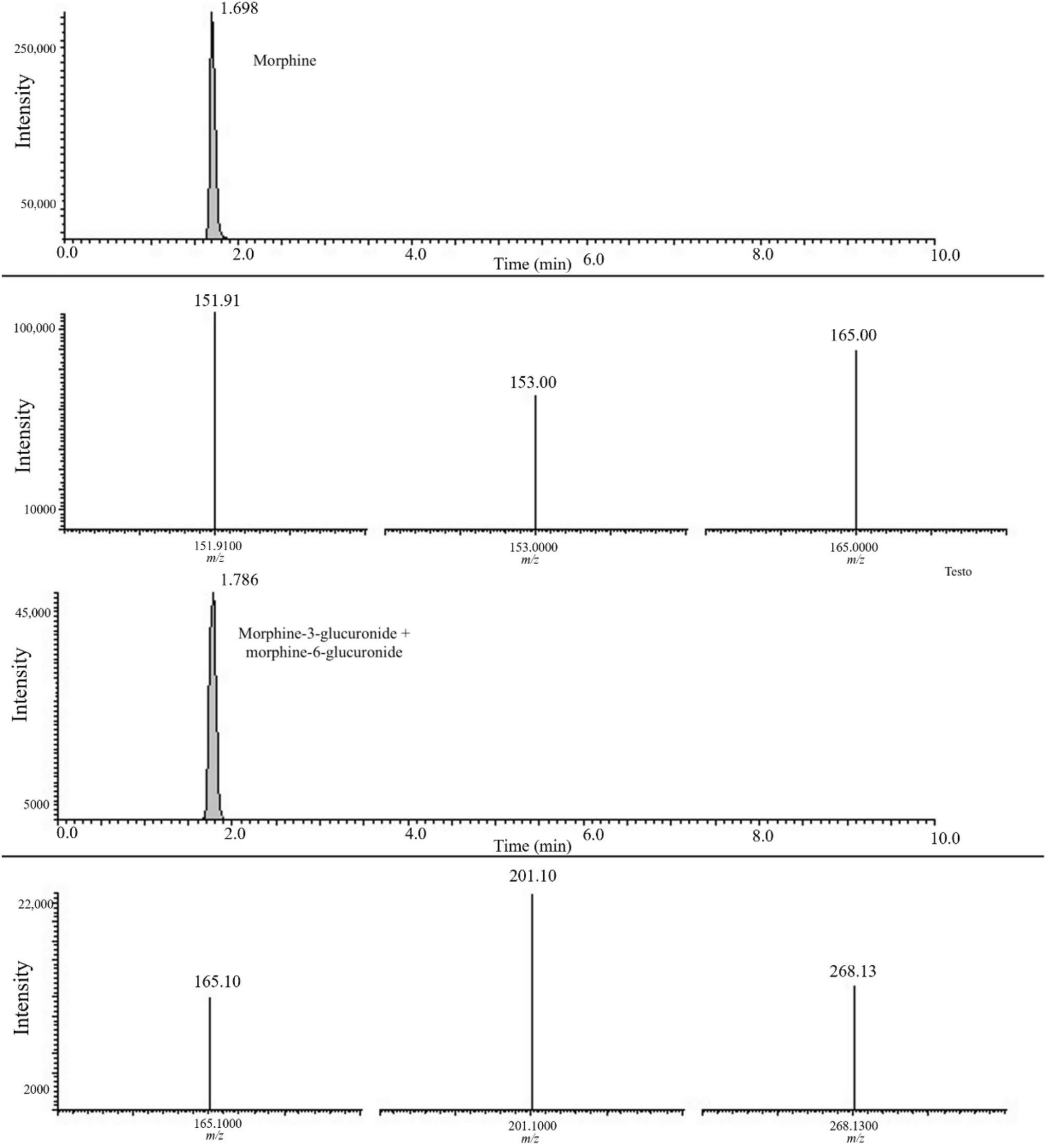
SRM chromatograms and their respective product ion spectra showing the analytes detected in a paraffin block embedded 20 years ago (case no. 2). SRM chromatograms: the top row, morphine; the third row, morphine-3-glucuronide + morphine-6-glucuronide

Results collected from 20-year-old toxicological reports and the paraffin-embedded samples showed a positivity for morphine and morphine-3-glucuronide plus morphine-6-glucuronide in all the cases considered (Table 3). 6-Acetylmorphine was detected in four cases (case nos. 3–5 in both samples, and case no. 2 only in an analytical report for the liver but missing in the old embedded sample). The SRM chromatogram of old embedded blocks (case no.2) highlighted the presence of morphine and morphine-3-glucuronide + morphine-6-glucuronide and relative fragmentation as shown in Fig. 2.

## Discussion

Liver was selected as the most appropriate matrix because this organ is of great toxicological interest; it is abundantly sampled during autopsy examination, and is always taken into consideration both for histological and toxicological investigations.

Regarding the extraction, isolation and purification of the analytes from these embedded matrices, we investigated several procedures to establish an effective method suitable for our purposes. While the grinding of the embedded samples into small pieces was a procedure in common with similar toxicological research articles [11, 12], the solubilization of the paraffin wax embedding the samples was a more delicate procedure. Classic deparaffinization involving the use of xylene or a xylene substitute and subsequent rehydrating passages in a decreasing alcoholic ladder [17] was considered as too invasive under a toxicological point of view; the washing out and subsequent loss of analytes had to be taken into consideration, and we therefore decided to proceed by solubilizing paraffin in a non-polar solvent *n*-hexane. Analytes of toxicological interest are polar molecules and we therefore decided to proceed with a liquid–liquid extraction using acetonitrile. *n*-Hexane and acetonitrile are not mixable solvents and solubilized paraffin remains in the non-polar phase; on the other hand, xenobiotics have a better chemical affinity for the polar phase. The subsequent separation of the two solvents and the exsiccation of acetonitrile granted us an efficient way for extracting and concentrating the molecules of interest.

In 2013, Vuori et al. [12] developed a new method for extracting analytes from FFPE samples. In this study, two cases, where paraffin-embedded samples were the only specimens available, underwent analysis and several drugs were detected (oxazepam, temazepam, trimethoprim and diazepam in one case and temazepam, citalopram, oxazepam, desmethyldiazepam, venlafaxine and its metabolite *o*-desmethylvenlafaxine in another case). However this study consisted of solely qualitative evaluations of the results. Another study [11], assessed in 2018, highlighted the presence of ajmaline in histological samples of liver and heart, but this study as well was evaluated only under a qualitative point of view and nothing about the details of the method was given.

Regarding our results following HPLC–MS/MS analysis and the subsequent quantitation, all selected cases analyzed were positive for morphine and morphine-3-glucuronide plus morphine-6-glucuronide, and three cases out of ten resulted in traces (values lower than LLOQ) of 6-acetylmorphine. This latter molecule seldom appeared in liver samples in heroin-related forensic cases, and the cases that were positive for 6-acetylmorphine (cases nos. 3, 4 and 7) were those with higher concentrations of morphine (Table 2).

Chemically and histologically treated identical liver samples, which were extracted with the previously discussed method, were also analyzed (Table 2). All the freshly embedded samples were also positive for the same substances except for 6-acetylmorphine, most of which were not detected except for the five matrices (shown as “Trace”). A ratio between the amounts of morphine detected in liver samples and in embedded samples revealed a quite constant value; the mean ratio was at 1.72 (liver/embedded: L/E) with the lowest ratio value at 1.61 and the highest at 1.82. A ratio between the amounts of morphine-3-glucuronide plus morphine-6-glucuronide detected in liver samples and in the embedded samples also revealed a quite constant value; the mean ratio was at 1.71 (liver/embedded) with the lowest ratio value at 1.63 and the highest at 1.81. A ratio for 6-acetylmorphine concentrations, of course, could not be calculated since the substance was present in traces only. Most probably, the embedding process and the extracting procedure led to reduction of the amounts of analytes, but still allowed satisfactory evaluation of the presence of morphine, morphine-3-glucuronide and morphine-6-glucuronide in histologically treated samples (more than 50% of the amounts extracted from corresponding raw liver matrices). The positivity of 6-acetylmorphine in about one of the three cases is probably caused by the extremely low concentrations of the analyte in the liver matrices, with a concentration below LOD.

Old toxicological analyses, assessed on liver samples (5 cases) (Table 3) screened in the year 2000, were all positive for heroin administration. Morphine, morphine-3-glucuronide and morphine-6-glucuronide were recorded to be detected in all liver samples, and 6-acetylmorphine was recorded in two out of five cases in traces, and in these cases opiates’ concentrations were high. Analysis of the records stored in the archives of the Institute of Legal Medicine, by the previously described procedure, gave positive results for the samples analyzed; morphine, morphine-3-glucuronide and morphine-6-glucuronide were detected in all embedded specimens, while 6-acetylmorphine was detected in two cases (case nos. 2 and 5).

A ratio between the amounts of morphine in the liver and in old embedded samples revealed a quite constant value (Table 3); the mean ratio was 1.52 (liver/old embedded samples) with the lowest ratio value at 1.38 and the highest at 1.61. A ratio between the amounts of morphine-3-glucuronide plus morphine-6-glucuronide detected in liver samples and in old embedded samples also revealed a quite constant value; the mean ratio was of 1.53 (liver/embedded samples) with the lowest ratio value at 1.44 and the highest at 1.60. The ratio calculations for 6-acetylmorphine concentrations could not be carried out, since the substance was “not detected” or, if present, only in traces.

When the current mean L/E ratio of FFPE samples is compared to that with FFPE 20 years ago, the current mean value 1.72 is slightly higher than the old mean value 1.52; this suggests that the old analysis of liver samples gave slightly lower result value due to lower recovery rates or higher suppressive matrix effects by the extraction and LC–MS/MS techniques conducted 20 years ago, while the target compounds kept in FFPE blocks were sufficiently stable during the 20 years.

## Conclusions

Our study focused on the feasibility of an efficient extracting procedure with the purpose of validating a new substrate to provide reliable toxicological data for cases where standard fresh samples were not available anymore, and the only substrates that can be analyzed are FFPE blocks. This work demonstrated the validity of a simple liquid–liquid extraction of target compound(s) from the FFPE block samples and confirmed the feasibility of these types of analysis.

Although further investigations should be carried out concerning different analytes, different visceral organs and different extracting methods, we are optimistic about coping with every type of samples, because hydrophilic metabolites of opiate could be quantitated with more than 50% of recovery (see the ratios in Tables 2 and 3). To our knowledge, this is the first paper describing the details of quantitation of target compounds in paraffin-embedded block as a new alternative matrix. It was surprising that the target compounds could be quantitated from FFPE blocks created as long as 20 years ago.
